# Spatial distribution of triatomines in domiciles of an urban area of the Brazilian Southeast Region

**DOI:** 10.1590/0074-02760150352

**Published:** 2016-01

**Authors:** João Victor Leite Dias, Dimas Ramon Mota Queiroz, Helen Rodrigues Martins, David Eladio Gorla, Herton Helder Rocha Pires, Liléia Diotaiuti

**Affiliations:** 1 Fundação Oswaldo Cruz, Centro de Pesquisas René Rachou, Laboratório de Triatomíneos e Epidemiologia da Doença de Chagas, Belo Horizonte, MG, Brasil; 2 Universidade Federal dos Vales do Jequitinhonha e Mucuri, Departamento de Farmácia, Laboratório de Doenças Parasitárias, Diamantina, MG, Brasil; 3 Consejo Nacional de Investigaciones Científicas y Técnicas, Instituto Multidisciplinario de Biología Vegetal, Córdoba, Argentina

**Keywords:** Triatominae, spatial analysis, vector control, urbanisation

## Abstract

Reports of triatomine infestation in urban areas have increased. We analysed the spatial distribution of infestation by triatomines in the urban area of Diamantina, in the state of Minas Gerais, Brazil. Triatomines were obtained by community-based entomological surveillance. Spatial patterns of infestation were analysed by Ripley’s K function and Kernel density estimator. Normalised difference vegetation index (NDVI) and land cover derived from satellite imagery were compared between infested and uninfested areas. A total of 140 adults of four species were captured (100 *Triatoma vitticeps*, 25*Panstrongylus geniculatus*, 8 *Panstrongylus megistus*, and 7 *Triatoma arthurneivai* specimens). In total, 87.9% were captured within domiciles. Infection by trypanosomes was observed in 19.6% of 107 examined insects. The spatial distributions of*T. vitticeps*, *P. geniculatus*, *T. arthurneivai*, and trypanosome-positive triatomines were clustered, occurring mainly in peripheral areas. NDVI values were statistically higher in areas infested by *T. vitticeps* and *P. geniculatus*. Buildings infested by these species were located closer to open fields, whereas infestations of *P. megistus* and*T. arthurneivai* were closer to bare soil. Human occupation and modification of natural areas may be involved in triatomine invasion, exposing the population to these vectors.

Chagas disease represents a major public health issue in Latin American countries. Human infection occurs mainly by vector-borne transmission, in which the protozoan*Trypanosoma cruzi* (Chagas 1909) is transmitted by infected triatomines (Hemiptera: Reduviidae) (Rassi Jr et al. 2010).

In Brazil, Chagas disease transmission has been associated with people living in rural areas and with poor housing conditions in which triatomines are able to colonise (Coura 2007). The species primarily involved in transmission of *T. cruzi* in Brazil during the XX century was the domestic insect *Triatoma infestans* (Klug 1834). This allochthonous species was found in domiciles throughout nearly all the endemic area, although other species of triatomine bugs were of primary importance in large areas of Brazil, especially *Triatoma brasiliensis* Neiva 1911 and *Panstrongylus megistus* (Burmeister 1835) (Dias 2007).

After a sustained vector control program, Brazil was certified as free from *T. cruzi* transmission by *T. infestans* in 2006*.* Nevertheless, native triatomine species are continuously observed invading and colonising artificial environments (Abad-Franch et al. 2013). The expansion of human-inhabited areas, including cities, may disturb sites where natural cycles of *T. cruzi* occur, leading triatomines to invade domiciles, and also maintaining synanthropic reservoirs close to dwellings (Coura 2007). In Brazil, reports of infestation by autochthonous triatomines in domiciles of urban areas have increased during recent years (Santana et al. 2011, Maeda et al. 2012, Carvalho et al. 2014, Rodrigues et al. 2014, Ribeiro Jr et al. 2015). The objective of this study was to evaluate the importance of this domestic invasion by triatomines and the spatial pattern of invasion occurrence in an urban scenario of the Southeast Region in Brazil.

## MATERIALS AND METHODS

*Study area* - This study was performed in the urban area of the municipality of Diamantina, located in the Jequitinhonha Valley region, in the northeast of the state of Minas Gerais, Brazil. This region was one of the most important areas in terms of Chagas disease transmission in Brazil (Dias et al. 1985). In the early 1980s, 11.7% of the rural population of the Diamantina municipality were infected by *T. cruzi* (MS/SUCAM/DIDOCh 1980).

The municipality has an area of 3,892 km^2^, and its population was estimated at 47,803 people in 2014 (MP/IBGE 2014).

Climate is classified as Cwb, according to the Köppen-Geiger climate classification system (Alvares et al. 2013), and exhibits two distinct seasons: a rainy season that occurs between October-March and a dry season that occurs between April-September. The annual average temperature is 19ºC and the annual precipitation is approximately 1,400 mm (Vieira et al. 2010).

Diamantina is located in the southern area of the municipality, at approximately 1,300 m above sea level, placed atop a plateau known as the Diamantina Plateau and surrounded by the Cristais Mountains, which are part of the Espinhaço Mountain Range. Diamantina is composed of 25 neighbourhoods. At least 31,654 people live in the city, which contains approximately 20,400 buildings (Diamantina 2009). In this area, soils are shallow and sandy, with a quartzite substrate. Vegetation is characterised by rocky fields known as *camposrupestres,* which are typical of the Espinhaço Mountain Range (Eiten 1992, Costa 2005, Vasconcelos 2011). Forest fragments are often found associated with watercourses (Diamantina 2009).

Since 1999, the central area of Diamantina has been recognised by the United Nations Educational, Scientific, and Cultural Organization as a World Heritage Site (UNESCO 2000), which implies the existence of specific laws for urban management, leading people to occupy peripheral areas of the city.

*Triatomine collection* - Triatomines were captured during the activities of entomologic surveillance of Chagas disease, between September 2011-August 2014. People who found a suspect insect in their houses sent it to the municipal health service where specific identification and a parasitological exam of triatomine faeces were performed using optical microscopy. When insects were confirmed to be triatomines, a public health agent visited the house and performed a full entomological evaluation according to the Southern Cone Initiative protocol (OPS 1993).

We verified the species identification of triatomine species and data associated with the insect collection was recorded (house address and geographic coordinates, name of householder, place where the triatomine was found, sex or nymph stage, positivity for trypanosome, and information about who captured the insect - whether it was captured by the house owner or a health professional).

*Spatial analysis* - Infested houses were geo-referenced with a handheld GPS unit (GPS Map 76S, Garmin™). The geographic coordinate recorded in front of the infested house was considered for analysis. A “shapefile” of the 25 urban neighbourhoods of Diamantina was drawn based on the Brazilian National Health Foundation sketches with the support of a Google Earth™ scene as a reference.

The pattern of the spatial distribution of different triatomine species and trypanosome-infected insects was evaluated using graphical analysis of univariate Ripley’s K-function expressed as an L function. The analysis identifies clustered, randomly, or regularly distributed events in an area; pattern significance may be evaluated by simulations based on complete spatial randomness (Dixon 2002). Visually identified hotspots were calculated by a Kernel density estimator, which is a nonparametric interpolation technique based on the occurrence of events over a region of interest and smoothed by a searching radius (Gatrell et al. 1996).

The spatial analysis of triatomine invasion events was carried out using the K-function included in SPRING 5.2.7 (Câmara et al. 1996), in which we considered distances between zero-1,000 m. Significance was evaluated with 999 simulations considering a significance level of 5%. A quartic Kernel density estimation function was calculated in TerraView 4.2.2 (dpi.inpe.br/terraview/) using an adaptive radius, which varies according to the number of events and the extent of the study area (Lagrotta et al. 2008).

We expected that vegetation cover would influence the occurrence of triatomine invasions because of the association between vegetation and the ecotopes of triatomines and their hosts. Vegetation cover [estimated from normalised density vegetation index (NDVI) imagery, see below] was compared between occurrence and nonoccurrence triatomine invasion sites. The coordinates of the infested houses served as the occurrence sites, whereas 150 random points generated over uninfested neighbourhoods served as the nonoccurrence sites.

The NDVI values were calculated from bands 4 (red - wavelength 0.636-0.673 µm) and 5 (near infrared - wavelength 0.851-0.879 µm) of the Operational Land Imager on the LANDSAT 8 satellite. Scene acquisition dates were 25 August 2013 and 28 August 2014 (earthexplorer.usgs.gov/). Atmospheric correction was performed using the dark object subtraction method (Chavez Jr 1988). NDVI values were calculated for each date using SPRING 5.2.7 (Câmara et al. 1996). Because vegetation could vary from year to year due to differences in rainfall, we calculated the difference in NDVI values between dates to evaluate its change throughout two consecutive years. Because the NDVI values showed little change between 2013-2014 (restricted to only a few changed pixels in the image), the most recent image was chosen to evaluate the NDVI in sites where triatomines were both found and not found.

To compare the NDVI values, buffers were drawn surrounding each occurrence and nonoccurrence points with radii of 50 m, 100 m, 150 m, 200 m, and 250 m. The NDVI average for each circle buffer was calculated and compared to triatomine occurrence and nonoccurrence points, by species.

The association between house invasion by triatomines and distance to land cover type was analysed. Land cover was classified into the following three classes according to NDVI values and based on field observations: bare soil (NDVI between 0-0.15), open fields (NDVI between 0.15-0.3), and forest (NDVI ≥ 0.3). Distances between infested or uninfested houses and land cover classes were measured and compared according to species.

A Kruskal-Wallis test followed by Dunn’s multiple-comparison tests, with a significance level of 0.05 (GraphPad Prism™ 5), was used to compare NDVI values with distances to the vegetation cover classes at both triatomine occurrence and nonoccurrence sites.

*Ethics* - This study was approved by the Ethical Committee of the Federal University of Jequitinhonha e Mucuri Valleys (protocol 520.250).

## RESULTS

A total of 140 adult triatomines of four species were captured between September 2011-August 2014 and referred to the Diamantina health services. *Triatoma vitticeps* (Stål 1859) was the most frequent (73♂, 27♀), followed by*Panstrongylus geniculatus* (Latreille 1811) (15♂, 10♀),*P. megistus* (6♂, 2♀), and *Triatoma arthurneivai* Lent & Martins 1940 (5♂, 2♀).

Among the captured triatomines, examination of infection in 33 insects was not possible because the specimens were dry and therefore did not present intestinal content for analysis. Of a total of 107 examined insects, none of the *P. megistus* (5 examined specimens) and *T. arthurneivai* (6 examined specimens) were infected; however, trypanosome infections were observed in*P. geniculatus* (5/13 examined specimens) and *T. vitticeps* (16/83 examined specimens), totalling 19.6% of the examined insects.

Most of the triatomines were captured inside houses (87.9%). Infested sites were primarily bedrooms (33.6%) and living rooms (32.1%), followed by bathrooms (6.4%), kitchens (5.7%), and utility areas (5.7%). Other locations included back yards (3.6%), balconies (0.7%), garages (2.1%), prison courtyard (0.7%), sports courts (0.7%), streets (2.9%), and walls (2.1%). Two cases in which insects (1.4%) were captured inside the houses lacked information about where the capture occurred and three (2.1%) triatomines had no site capture information. Infected *P. geniculatus* were captured in kitchens (2), living rooms (2), and utility areas (1), whereas infected *T. vitticeps* were captured in bedrooms (6), living rooms (4), walls (2), balconies (1), bathrooms (1), utility areas (1), and streets (1).

**Fig. 1:**
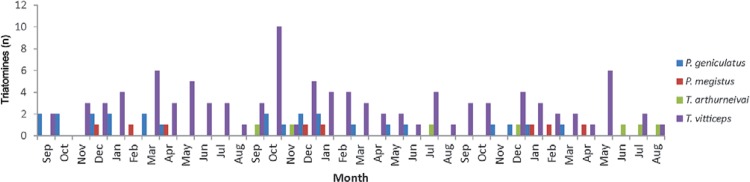
: number of triatomines by species captured in urban area of Diamantina, state of Minas Gerais, Brazil, September 2011-August 2014, according to month.

Captures occurred in all months, but were more frequent in December and January (Fig. 1). Only one specimen was captured by the public health services agent; all other specimens were captured by house owners who subsequently notified public health. There were 114 infested houses, largely distributed (72%) among 18 neighbourhoods. In 15 (13.2%) houses, multiple episodes of infestation occurred - twice in eight houses, three times in five houses, four times in one house, and six times in one house. In five houses, triatomines of two different species were captured - *T. vitticeps*and *P. geniculatus*. Among the infested houses, 112 (98.3%) were georeferenced, but two had incomplete addresses, which precluded house finding and geo-referencing. All the infested houses were made of brick and had roofs with slab and/or ceramic or asbestos tiles.

**Fig. 2:**
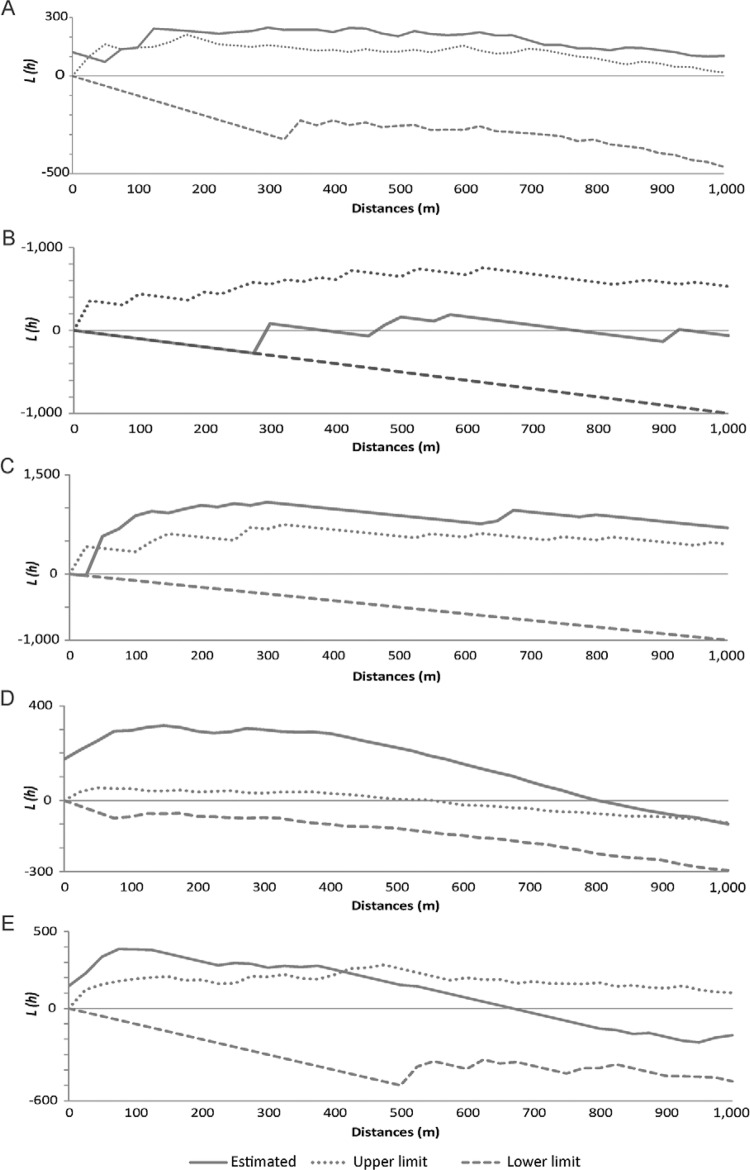
: K-function expressed as L-function values according to distances between buildings infested by triatomines in the city of Diamantina, state of Minas Gerais, Brazil, September 2011-August 2014. A:*Panstrongylus geniculatus*; B: *Panstrongylus megistus*; C: *Triatoma arthurneivai*; D:*Triatoma vitticeps*; E: triatomines infected by trypanosomatids.

**Fig. 3:**
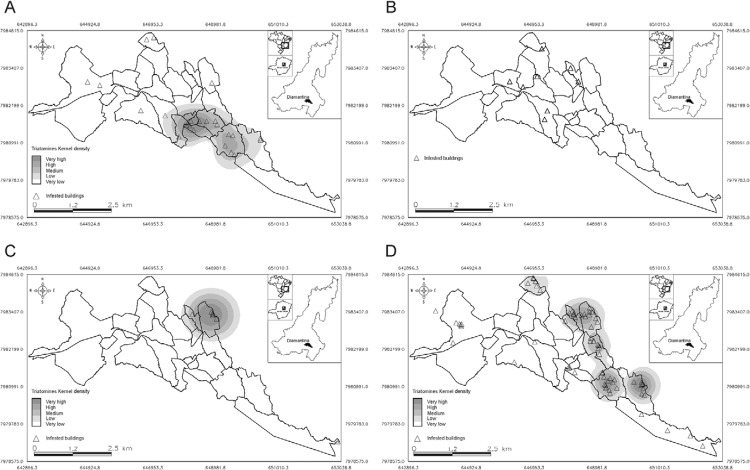
: spatial distribution of triatomines captured in the city of Diamantina, state of Minas Gerais, Brazil, September 2011-August 2014. A: *Panstrongylus geniculatus*; B: *Panstrongylus megistus*; C: *Triatoma arthurneivai*; D:*Triatoma vitticeps*.

Regarding the spatial distribution of infested houses, only *P. megistus* did not present significant clustered distribution patterns by K-function analysis (Fig. 2A-D). Positivity for trypanosomes was clustered as well (Fig. 2E). The distributions of *P. geniculatus*, *T. arthurneivai*, and *T. vitticeps* exhibited an evident peripheral pattern (Fig. 3).

**Fig. 4:**
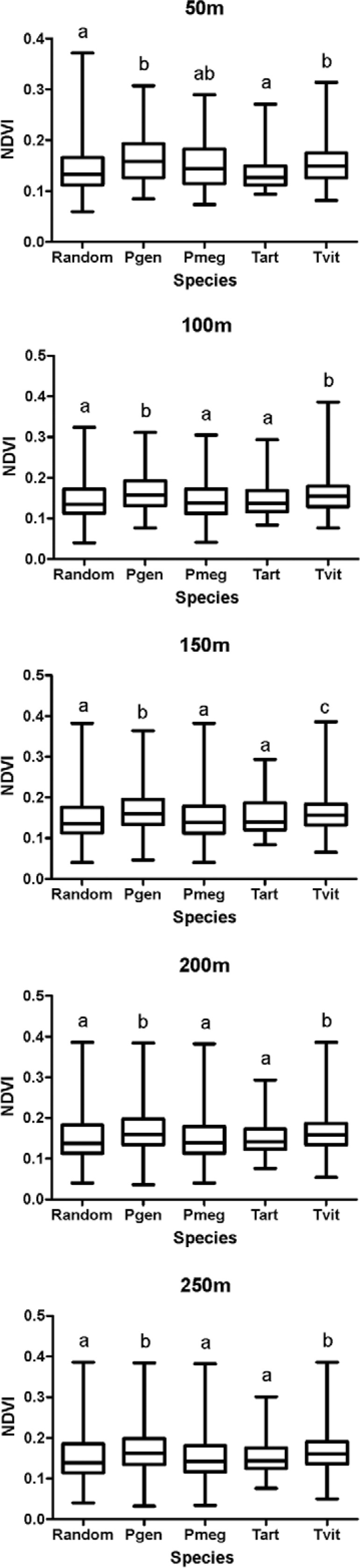
: normalized density vegetation index (NDVI) around triatomine-infested buildings and “uninfested” areas in the city of Diamantina, state of Minas Gerais, Brazil, according to radius surrounding infested and “uninfested” sites. Different letters represent significant statistical differences (p ≤ 0.05) between groups in the Kruskal-Wallis test. Pgen: *Panstrongylus geniculatus*; Pmeg: *Panstrongylusmegistus*; random: randomized points in uninfested areas; Tart: *Triatoma arthurneivai*; Tvit:*Triatoma vitticeps*.

NDVI values were significantly higher in areas infested by *P. geniculatus* and *T. vitticeps* than in areas infested by other species or uninfested. No significant differences were observed between the NDVI values for *P. megistus* and *T. arthurneivai*infested areas and uninfested areas (Fig. 4).

**Fig. 5:**
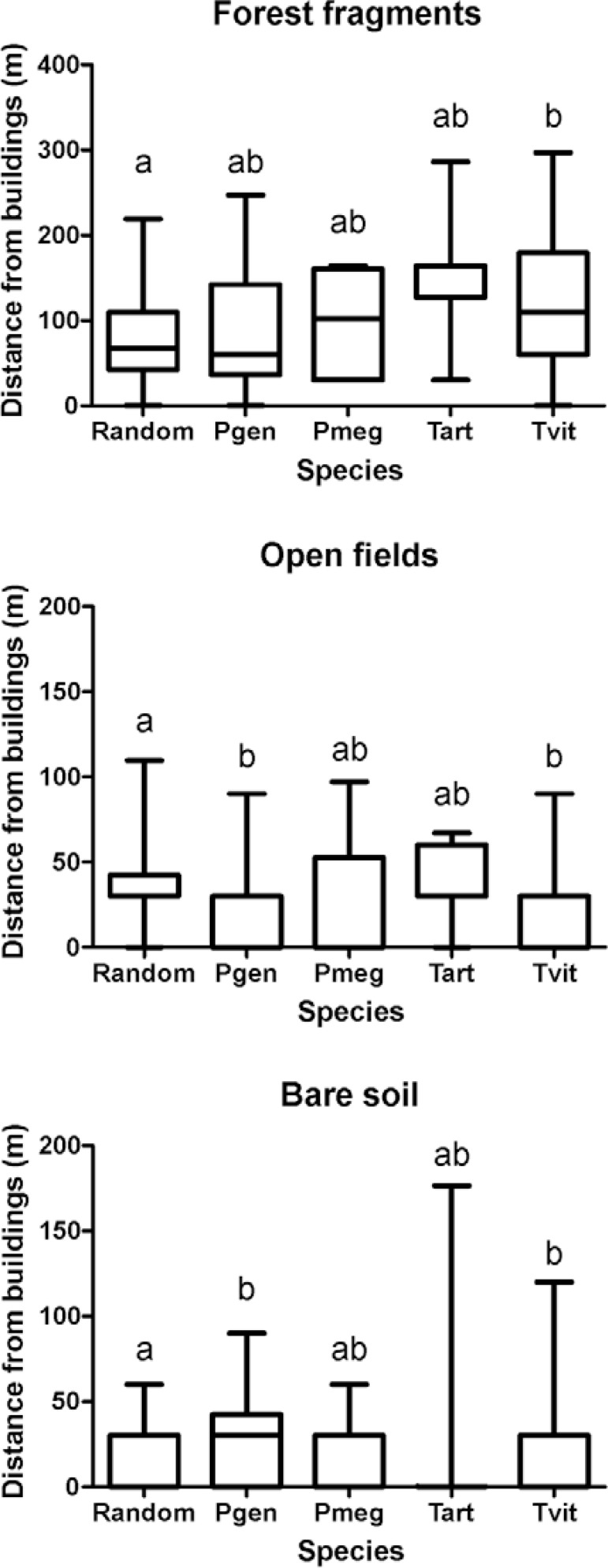
: distances from triatomine-infested buildings and “uninfested” points to areas with distinct land cover in the city of Diamantina, state of Minas Gerais, Brazil. Different letters represent significant statistical differences (p ≤ 0.05) between groups in the Kruskal-Wallis test. Pgen: *Panstrongylus geniculatus*; Pmeg:*Panstrongylusmegistus*; random: randomized points in uninfested areas; Tart: *Triatoma arthurneivai*; Tvit:*Triatoma vitticeps*.

Considering land cover, the area exhibited a highly heterogeneous pattern. However, some triatomine species were observed closer to specific land cover than others. In contrast to other species, houses infested by *T. vitticeps* were further away from forest fragments than uninfested areas. Houses infested by*T. vitticeps* and *P. geniculatus* were located significantly closer to open fields than uninfested random points. For these two species, an inverse trend was observed in areas surrounded by bare soil (Fig. 5).

## DISCUSSION

Although originally associated with rural areas, reports of infestation by triatomines are increasing in urban areas, including those species known to be involved in *T. cruzi* transmission to humans (Guzman-Tapia et al. 2007, Santana et al. 2011, Maeda et al. 2012, Carvalho et al. 2014, Rodrigues et al. 2014, Ribeiro Jr et al. 2015).

Winged adult specimens of four triatomine species were captured throughout the year in the domiciles of an urban area of Diamantina. Two of these species were found naturally infected by trypanosomes. It is possible to infer that these trypanosomes were *T. cruzi*, what is supported by Reis et al. (2013), which detected kDNA of this parasite in faeces of triatomines from this region, including some of the insects in the present study.

*T. vitticeps* was the most frequently captured triatomine in Diamantina and was highly infected by trypanosomes, as also observed in other parts of Brazil (Dias et al. 1989, dos Santos et al. 2005, 2006a, 2014, Souza et al. 2010). Even with its low vector capability (dos Santos et al. 2006b), some reports incriminate the species in the transmission of *T. cruzi* to humans (Lorosa et al. 2003, 2008, Sangenis et al. 2015), pointing out the importance of a close entomological surveillance on this species in its areas of occurrence.

In contrast to findings from other areas (Dias et al. 1989, Gonçalves et al. 1998, 2000, Leite et al. 2010), more males than females of *T. vitticeps* were captured in Diamantina*.* This discrepancy may be due to population differences driven by environmental characteristics that may influence insect dispersal. Gürtler et al. (2014) observed an influence of weight/length ratio on the flight capability of females of *T. infestans* that was not observed for males. In addition to this, these authors demonstrated that females, in sites with constant food availability, were less prone to fly, and this behaviour may be associated with the maintenance of a high weight/length ratio. Most findings of *T. vitticeps* are reported from areas covered by the Atlantic Forest [states of Espírito Santo (ES) and Rio de Janeiro], but Diamantina is placed within a *Cerrado*biome, surrounded by rocky fields.

The spatial distribution of infestation by *T. vitticeps* in Diamantina was clustered and was mainly observed in peripheral neighbourhoods. These areas represent the boundary between the urban area and the Cristais Mountains, a preserved area mainly covered by *campos rupestres*. This condition may explain the fact that domiciles infested by *T. vitticeps* were closer to open-fields than uninfested random points. The terrain in this area is irregular, exhibiting fissured rocks. Notably, Leite et al. (2010) observed that domiciliary infestation by *T. vitticeps* is associated with areas where the terrain is highly variable, in which crevices might be the shelter for triatomine hosts in ES.

Higher NDVI values close to *T. vitticeps* infested domiciles (even when those houses were far from forest fragments) might be associated with border areas with a low building density, so that circles around infested houses would include a smaller area lacking vegetation.

Although *P. geniculatus* is considered a sylvatic triatomine, it was observed colonising pigsties in Brazil (Valente et al. 1998) and was involved in oral acute Chagas disease urban outbreaks in Venezuela (Alarcón de Noya et al. 2010,Muñoz-Calderón et al. 2013) and vectorial transmission of *T. cruzi* in Peru (Vega et al. 2006). The high infection rates observed for this triatomine may be explained by its association with important hosts of *T. cruzi*, particularly armadillos (Chagas 1912, Martins et al. 1940, Barretto 1979, Alvarado-Otegui et al. 2012). Species distribution in Diamantina was more conspicuous in areas close to open-fields and forest fragments that may be the natural foci of these species.

*P. megistus* is the most important *T. cruzi* vector in the vast areas of Brazil. In the urban area of Diamantina, this triatomine was randomly distributed in areas with low vegetation cover as estimated by NDVI values. However, it is worth remarking that isolated patches with high NDVI values were found next to houses infested by *P. megistus*. These patches may represent small forest fragments that would maintain the sylvatic foci of *P. megistus*, where adults could disperse from, as observed by Santos Jr et al. (2013), into other urban areas of Brazil. Flight represents the main dispersal mechanism for triatomines and, as observed for *T. infestans* and*Triatoma sordida*, these insects can easily span distances greater than 100 m (Schofield et al. 1991,^1992^).

*T. arthurneivai* is a rarely captured species, reported only in areas covered by *campos rupestres*, located in the southern part of the Espinhaço Mountain Range (Lent & Martins 1940, Dias et al. 2011). Based on its restricted distribution and singular characteristics of vegetation and relief,Dias et al. (2011) proposed that the Espinhaço Mountain Range is the endemic area of this triatomine. The present study results agreed with these previous observations; infestations by *T. arthurneivai* were clustered in hotspots of occurrence found near borders between the urban area and the Cristais Mountains, a segment of the Espinhaço Mountain range.

Despite the success in eliminating domestic populations of triatomines, the Chagas disease control services of Brazil face challenges in maintaining the entomologic surveillance. Although most triatomines captured in Diamantina domiciles have low colonisation capacity in houses, their high prevalence of trypanosome infections may represent a considerable risk for the transmission of *T. cruzi* to humans. Thus, in areas such as Diamantina, where there is a mosaic of urban areas and naturally preserved environments that may function as “dispersive islands”, strengthening entomological surveillance efforts in these scenarios is needed.
